# Supplementary material for: Inequality of opportunity in selection procedures limits diversity in higher education: An intersectional study of Dutch selective higher education programs
Source: PLoS One. 2023 Oct 13;18(10):e0292805. doi: 10.1371/journal.pone.0292805 (PMC10575509; doi:10.1371/journal.pone.0292805)
Supplement: S1 Table — (DOCX) [file pone.0292805.s001.docx]

# **S1: Clusters of study programs**

**HBO programs (HBO = Higher Professional Education) – 19 study programs**

| **Cluster** | **HBO program** | **Number of HBOs using selection (included in this research)** | **Number of HBOs not using selection (not included in this research)** |
| --- | --- | --- | --- |
| **SH1** | Allied Medical Care | 3 | 0 |
|  | Nursing | 1 | 16 |
|  | Midwifery | 3 | 0 |
| **SH2** | Dental Hygiene | 4 | 0 |
|  | Denturism | 1 (2020) | 0 |
|  | Optometry | 1 | 0 |
| **SH3** | Biology and Medical Laboratory Research | 2 (2019), 1 (2020) | 10 |
|  | Forensic Science | 2 | 0 |
|  | Medical Imaging and Radiation Therapy | 1 | 2 |
| **SH4** | Physiotherapy | 6 | 4 |
|  | Psychomotoric Therapy/Psychomotricity | 1 | 0 |
|  | Sport Studies | 1 | 7 |
| **SH5** | Creative Media and Game Technologies | 1 | 4 |
|  | Fashion & Textile Technologies; | 1 | 1 |
|  | Industrial Design Engineering | 1 | 6 |
|  | Art and Economics | 1 (2020) | 0 |
| **SH6** | Applied Psychology | 4 | 2 |
|  | Applied Biology | 1 | 1 |
|  | Skin Therapy | 2 | 0 |

**University programs – 28 study programs**

| **Cluster** | **University program** | **Number of universities using selection (included in this research)** | **Number of universities not using selection (not included in this research)** |
| --- | --- | --- | --- |
|  |  |  |  |
| **SU1** | Medicine | 8 | 0 |
| **SU2** | Dentistry | 4 | 0 |
|  | Pharmacy | 1 | 1 |
| **SU3** | Psychobiology | 1 | 0 |
|  | Psychology | 6 (2019), 9 (2020) | 2 |
| **SU4** | Biomedical Sciences | 4 | 2 |
|  | Biomedical Engineering | 1 (2019) | 2 |
|  | Clinical Technology | 2 | 0 |
| **SU5** | Biology | 1 | 0 |
|  | Biotechnology | 1 | 0 |
|  | Nutrition and Health | 1 | 0 |
|  | Veterinary Medicine | 1 | 0 |
|  | Nanobiology | 1 | 0 |
| **SU6** | Artificial Intelligence | 4 | 2 |
|  | Industrial Design | 2 | 1 |
| **SU7** | Architecture, Urbanism & Building Sciences | 2 | 0 |
|  | Mechanical Engineering | 1 | 2 |
|  | Aerospace Engineering | 1 | 0 |
|  | Computer Science & Engineering | 2 | 2 |
|  | Global Sustainability Science | 1 | 0 |
| **SU8** | Business Administration | 1 (2019) | 0 |
|  | International Business | 2 | 1 |
|  | International Business Administration | 1 | 3 |
|  | Tax Law | 1 | 5 |
|  | Industrial Engineering & Management Science | 1 | 2 |
| **SU9** | International Relations and International Organization | 1 | 0 |
|  | Political Science | 1 | 3 |
|  | Criminology | 3 | 0 |
