# Supplementary material for: Inequality of opportunity in selection procedures limits diversity in higher education: An intersectional study of Dutch selective higher education programs
Source: PLoS One. 2023 Oct 13;18(10):e0292805. doi: 10.1371/journal.pone.0292805 (PMC10575509; doi:10.1371/journal.pone.0292805)
Supplement: S2 Table — *p < .05; **p<0.01; ***p<0.001. Ref. = reference category; PSVA = Primary, Secondary or Vocational or Agrarian; TMSDI: Turkish, Moroccan, Surinamese, Dutch Caribbean, or Indonesian; Urbanity Degree of postal code of residential address in 2015. Results in bold are statistically significant (p < .05). (DOCX) [file pone.0292805.s002.docx]

# **S2: Results from the univariable logistic regression for each cluster, performed on applicants eligible for placement**

|  | **TOTAL DATASET** | **SH1_2019** | **SH1_2020** | **SH2_2019** | **SH2_2020** |
| --- | --- | --- | --- | --- | --- |
|  |  | **Allied Medical Care; Nursing; Midwifery** | **Allied Medical Care; Nursing; Midwifery** | **Dental Hygiene, Denturism, Optometry** | **Dental Hygiene, Denturism, Optometry** |
|  | **OR (95% CI)** | **OR (95% CI)** | **OR (95% CI)** | **OR (95% CI)** | **OR (95% CI)** |
| **GENDER** |  |  |  |  |  |
| Female (ref. male) | **0.882 (0.857-0.908)***** | **1.978 (1.541-2.539)***** | **1.917 (1.495-2.457)***** | 1.031 (0.771-1.379) | 0.948 (0.703-1.28) |
| **AGE CATEGORY** |  |  |  |  |  |
| Year of birth 2000-2001 (ref. 1999 or earlier) | **1.161 (1.122-1.202)***** | **0.806 (0.662-0.982)*** | 0.784 (0.608-1.01) | **1.456 (1.165-1.819)**** | 0.947 (0.734-1.22) |
| Year of birth 2002 or later (ref. 1999 or earlier) | **1.108 (1.064-1.154)***** | **0.544 (0.417-0.71)***** | **0.585 (0.459-0.746)***** | 0.933 (0.625-1.391) | 1.135 (0.859-1.499) |
| **INCOME CATEGORY** |  |  |  |  |  |
| Percentile 71-90 (ref. 1-70) | **1.471 (1.407-1.537)***** | 1.258 (0.984-1.607) | **1.569 (1.213-2.03)**** | **1.691 (1.305-2.191)***** | **1.454 (1.116-1.895)**** |
| Percentile 91-100 (ref. 1-70) | **1.605 (1.542-1.671)***** | 1.045 (0.82-1.332) | 1.157 (0.899-1.489) | **2.572 (1.952-3.389)***** | **2.12 (1.607-2.796)***** |
| **ASSETS CATEGORY** |  |  |  |  |  |
| Percentile 41-80 (ref. 1-40) | **1.584 (1.517-1.654)***** | **1.824 (1.419-2.345)***** | **1.603 (1.248-2.057)***** | **1.761 (1.365-2.271)***** | **2.428 (1.87-3.154)***** |
| Percentile 81-100 (ref. 1-40) | **1.791 (1.715-1.871)***** | **1.836 (1.404-2.401)***** | **1.625 (1.241-2.128)***** | **3.02 (2.25-4.056)***** | **3.111 (2.291-4.225)***** |
| **NR. OF PARENTS ON WELFARE** |  |  |  |  |  |
| 1 or 2 parents (ref. 0) | **0.411 (0.383-0.442)***** | **0.376 (0.224-0.633)***** | **0.568 (0.338-0.954)*** | **0.484 (0.354-0.662)***** | **0.459 (0.334-0.63)***** |
| **NR. OF PARENTS WITH ANOTHER SOCIAL SERVICES INCOME (excl. welfare)** |  |  |  |  |  |
| 1 or 2 parents (ref. 0) | **0.715 (0.677-0.755)***** | **0.696 (0.503-0.965)*** | 0.991 (0.71-1.384) | 1.007 (0.724-1.4) | 0.726 (0.509-1.035) |
| **NR. OF PARENTS WITH BIG-REGISTRATION** |  |  |  |  |  |
| 1 or 2 parents (ref. 0) | 0.996 (0.955-1.039) | **1.877 (1.437-2.451)***** | **1.368 (1.051-1.78)*** | **2.755 (1.77-4.288)***** | **2.906 (1.863-4.534)***** |
| **NR. OF PARENTS WORKING IN PSVA EDUCATION** |  |  |  |  |  |
| 1 or 2 parents (ref. 0) | **1.061 (1.006-1.119)*** | **1.458 (1.04-2.046)*** | 1.148 (0.828-1.591) | 1.128 (0.699-1.822) | 0.976 (0.545-1.746) |
| **MIGRATION BACKGROUND** |  |  |  |  |  |
| TMSDI (ref. no migration background) | **0.506 (0.483-0.531)***** | **0.194 (0.133-0.282)***** | **0.298 (0.208-0.428)***** | **0.363 (0.276-0.477)***** | **0.328 (0.245-0.44)***** |
| Other (ref. no migration background) | **1.113 (1.078-1.149)***** | **0.547 (0.403-0.744)***** | **0.496 (0.36-0.683)***** | **0.404 (0.313-0.521)***** | **0.426 (0.33-0.551)***** |
| **URBANITY DEGREE** |  |  |  |  |  |
| Average (ref. (very) strongly urban) | **1.134 (1.088-1.181)***** | **2.02 (1.561-2.614)***** | **1.55 (1.193-2.014)**** | **1.818 (1.358-2.434)***** | **1.343 (1.004-1.797)*** |
| Weakly urban/not urban (ref. (very) strongly urban) | **1.202 (1.161-1.244)***** | **3.121 (2.532-3.846)***** | **2.83 (2.287-3.502)***** | **1.65 (1.276-2.133)***** | **1.488 (1.146-1.931)**** |

p<.05; **p<0.01; ***p<0.001

Ref. = reference category; PSVA = Primary, Secondary, Vocational or Agrarian; TMSDI: Turkish, Moroccan, Surinamese, Dutch Caribbean, or Indonesian; Urbanity Degree of postal code of residential address in 2015. Results in **bold** are statistically significant (p<.05).

|  | **SH3_2019** | **SH3_2020** | **SH4_2019** | **SH4_2020** | **SH5_2019** |
| --- | --- | --- | --- | --- | --- |
|  | **Biology and Medical Laboratory Research; Forensic Science; Medical Imaging and Radiation Therapy** | **Biology and Medical Laboratory Research; Forensic Science; Medical Imaging and Radiation Therapy** | **Physiotherapy; Psychomotoric Therapy/ Psychomotricity; Sport Studies** | **Physiotherapy; Psychomotoric Therapy/ Psychomotricity; Sport Studies** | **Creative Media and Game Technologies; Fashion & Textile Technologies; Industrial Design Engineering; Art and Economics** |
|  | **OR (95% CI)** | **OR (95% CI)** | **OR (95% CI)** | **OR (95% CI)** | **OR (95% CI)** |
| **GENDER** |  |  |  |  |  |
| Female (ref. male) | 0.855 (0.638-1.147) | 0.924 (0.688-1.241) | **1.201 (1.005-1.435)*** | **1.452 (1.23-1.713)***** | **2.662 (2.136-3.317)***** |
| **AGE CATEGORY** |  |  |  |  |  |
| Year of birth 2000-2001 (ref. 1999 or earlier) | 0.94 (0.677-1.304) | 1.077 (0.694-1.671) | 1.036 (0.85-1.264) | 0.898 (0.711-1.135) | 0.825 (0.658-1.033) |
| Year of birth 2002 or later (ref. 1999 or earlier) | 0.734 (0.508-1.058) | 0.872 (0.58-1.311) | 0.99 (0.765-1.281) | 0.933 (0.745-1.168) | 0.752 (0.516-1.095) |
| **INCOME CATEGORY** |  |  |  |  |  |
| Percentile 71-90 (ref. 1-70) | 1.036 (0.732-1.467) | 0.743 (0.509-1.084) | **1.576 (1.22-2.036)***** | 1.142 (0.889-1.468) | 0.831 (0.593-1.166) |
| Percentile 91-100 (ref. 1-70) | 0.98 (0.692-1.388) | 0.713 (0.493-1.031) | **1.766 (1.376-2.266)***** | 1.138 (0.894-1.448) | 1.081 (0.781-1.497) |
| **ASSETS CATEGORY** |  |  |  |  |  |
| Percentile 41-80 (ref. 1-40) | 1.06 (0.75-1.499) | 0.717 (0.501-1.026) | 1.041 (0.787-1.377) | 1.11 (0.849-1.451) | 1.228 (0.89-1.693) |
| Percentile 81-100 (ref. 1-40) | 0.864 (0.592-1.262) | **0.631 (0.424-0.938)*** | 1.321 (0.981-1.777) | 1.13 (0.854-1.495) | **1.584 (1.128-2.226)**** |
| **NR. OF PARENTS ON WELFARE** |  |  |  |  |  |
| 1 or 2 parents (ref. 0) | 0.875 (0.471-1.625) | 1.393 (0.739-2.624) | 0.834 (0.463-1.504) | 0.901 (0.46-1.762) | 0.987 (0.479-2.031) |
| **NR. OF PARENTS WITH ANOTHER SOCIAL SERVICES INCOME** |  |  |  |  |  |
| 1 or 2 parents (ref. 0) | 1.093 (0.711-1.68) | 1.129 (0.699-1.823) | **0.69 (0.485-0.982)*** | 0.755 (0.543-1.05) | 1.388 (0.899-2.144) |
| **NR. OF PARENTS WITH BIG-REGISTRATION** |  |  |  |  |  |
| 1 or 2 parents (ref. 0) | 0.92 (0.619-1.367) | 0.899 (0.611-1.324) | 1.136 (0.879-1.467) | **1.407 (1.096-1.808)**** | 1.44 (0.952-2.178) |
| **NR. OF PARENTS WORKING IN PSVA EDUCATION** |  |  |  |  |  |
| 1 or 2 parents (ref. 0) | 0.97 (0.605-1.555) | 1.264 (0.789-2.027) | 0.987 (0.735-1.325) | 0.976 (0.752-1.267) | 1.192 (0.795-1.788) |
| **MIGRATION BACKGROUND** |  |  |  |  |  |
| TMSDI (ref. no migration background) | 0.845 (0.556-1.283) | 1.557 (0.969-2.5) | 1.061 (0.682-1.65) | 0.912 (0.609-1.365) | 0.683 (0.459-1.015) |
| Other (ref. no migration background) | 1.106 (0.741-1.65) | 1.408 (0.952-2.084) | 0.849 (0.656-1.098) | 0.797 (0.631-1.006) | **0.736 (0.58-0.932)*** |
| **URBANITY DEGREE** |  |  |  |  |  |
| Average (ref. (very) strongly urban) | **1.702 (1.194-2.426)**** | 1.315 (0.924-1.872) | 0.833 (0.646-1.074) | 1.012 (0.797-1.286) | 0.956 (0.702-1.301) |
| Weakly urban/not urban (ref. (very) strongly urban) | **2.134 (1.586-2.872)***** | **1.474 (1.104-1.968)**** | **0.679 (0.549-0.839)***** | **0.761 (0.629-0.92)**** | 1.176 (0.886-1.562) |

*p<.05; **p<0.01; ***p<0.001

Ref. = reference category; PSVA = Primary, Secondary or Vocational or Agrarian; TMSDI: Turkish, Moroccan, Surinamese, Dutch Caribbean, or Indonesian; Urbanity Degree of postal code of residential address in 2015. Results in **bold** are statistically significant (p<.05).

|  | **SH5_2020** | **SH6_2019** | **SH6_2020** | **SU1_2019** | **SU1_2020** |
| --- | --- | --- | --- | --- | --- |
|  | **Creative Media and Game Technologies; Fashion & Textile Technologies; Industrial Design Engineering; Art and Economics** | **Applied Psychology; Applied Biology; Skin Therapy** | **Applied Psychology; Applied Biology; Skin Therapy** | **Medicine** | **Medicine** |
|  | **OR (95% CI)** | **OR (95% CI)** | **OR (95% CI)** | **OR (95% CI)** | **OR (95% CI)** |
| **GENDER** |  |  |  |  |  |
| Female (ref. male) | **1.657 (1.318-2.084)***** | **0.477 (0.394-0.579)***** | **0.753 (0.632-0.896)**** | **1.204 (1.085-1.337)***** | 1.036 (0.936-1.146) |
| **AGE CATEGORY** |  |  |  |  |  |
| Year of birth 2000-2001 (ref. 1999 or earlier) | 1.155 (0.876-1.522) | **1.245 (1.067-1.453)**** | 1.05 (0.883-1.249) | **1.161 (1.041-1.294)**** | 1.015 (0.879-1.174) |
| Year of birth 2002 or later (ref. 1999 or earlier) | 0.96 (0.709-1.298) | **1.611 (1.269-2.045)***** | **1.242 (1.038-1.486)*** | **1.944 (1.369-2.759)***** | **1.259 (1.087-1.457)**** |
| **INCOME CATEGORY** |  |  |  |  |  |
| Percentile 71-90 (ref. 1-70) | 1.045 (0.74-1.476) | **1.655 (1.375-1.992)***** | **1.507 (1.257-1.807)***** | **1.248 (1.066-1.462)**** | **1.328 (1.137-1.55)***** |
| Percentile 91-100 (ref. 1-70) | 1.207 (0.876-1.665) | **1.992 (1.661-2.39)***** | **1.536 (1.289-1.832)***** | **1.56 (1.358-1.791)***** | **1.508 (1.318-1.725)***** |
| **ASSETS CATEGORY** |  |  |  |  |  |
| Percentile 41-80 (ref. 1-40) | **1.461 (1.048-2.036)*** | **1.688 (1.408-2.025)***** | **1.512 (1.269-1.802)***** | 1.153 (0.994-1.337) | **1.44 (1.245-1.666)***** |
| Percentile 81-100 (ref. 1-40) | **1.58 (1.112-2.245)*** | **2.217 (1.815-2.71)***** | **1.804 (1.483-2.195)***** | **1.569 (1.359-1.812)***** | **1.647 (1.426-1.902)***** |
| **NR. OF PARENTS ON WELFARE** |  |  |  |  |  |
| 1 or 2 parents (ref. 0) | 0.525 (0.269-1.024) | **0.473 (0.358-0.623)***** | **0.504 (0.383-0.663)***** | **0.552 (0.436-0.699)***** | **0.622 (0.498-0.778)***** |
| **NR. OF PARENTS WITH ANOTHER SOCIAL SERVICES INCOME** |  |  |  |  |  |
| 1 or 2 parents (ref. 0) | 1.059 (0.682-1.645) | **0.672 (0.538-0.841)***** | 1.032 (0.825-1.29) | **0.764 (0.635-0.918)**** | 0.84 (0.7-1.008) |
| **NR. OF PARENTS WITH BIG-REGISTRATION** |  |  |  |  |  |
| 1 or 2 parents (ref. 0) | 1.593 (0.989-2.565) | **1.48 (1.164-1.88)**** | **1.361 (1.084-1.709)**** | **1.207 (1.071-1.36)**** | **1.187 (1.054-1.337)**** |
| **NR. OF PARENTS WORKING IN PSVA EDUCATION** |  |  |  |  |  |
| 1 or 2 parents (ref. 0) | 1.14 (0.751-1.731) | **1.765 (1.293-2.41)***** | 1.161 (0.878-1.535) | 1.009 (0.847-1.202) | 1.056 (0.887-1.257) |
| **MIGRATION BACKGROUND** |  |  |  |  |  |
| TMSDI (ref. no migration background) | **0.473 (0.324-0.69)***** | **0.456 (0.37-0.563)***** | **0.468 (0.383-0.573)***** | **0.579 (0.491-0.683)***** | **0.62 (0.53-0.726)***** |
| Other (ref. no migration background) | 0.914 (0.707-1.182) | **0.583 (0.478-0.71)***** | **0.642 (0.532-0.774)***** | 0.968 (0.858-1.091) | 0.969 (0.864-1.087) |
| **URBANITY DEGREE** |  |  |  |  |  |
| Average (ref. (very) strongly urban) | 0.936 (0.673-1.302) | **1.312 (1.072-1.605)**** | **1.278 (1.056-1.547)*** | 1.03 (0.9-1.179) | 1.034 (0.905-1.181) |
| Weakly urban/not urban (ref. (very) strongly urban) | 0.795 (0.594-1.064) | **1.233 (1.045-1.454)*** | **1.427 (1.211-1.682)***** | 1.03 (0.918-1.154) | **1.147 (1.025-1.285)*** |

*p<.05; **p<0.01; ***p<0.001

Ref. = reference category; PSVA = Primary, Secondary or Vocational or Agrarian; TMSDI: Turkish, Moroccan, Surinamese, Dutch Caribbean, or Indonesian; Urbanity Degree of postal code of residential address in 2015. Results in **bold** are statistically significant (p<.05).

|  | **SU2_2019** | **SU2_2020** | **SU3_2019** | **SU3_2020** | **SU4_2019** |
| --- | --- | --- | --- | --- | --- |
|  | **Dentistry; Pharmacy** | **Dentistry; Pharmacy** | **Psychobiology; Psychology** | **Psychobiology; Psychology** | **Biomedical Sciences; Biomedical Engineering; Clinical Technology** |
|  | **OR (95% CI)** | **OR (95% CI)** | **OR (95% CI)** | **OR (95% CI)** | **OR (95% CI)** |
| **GENDER** |  |  |  |  |  |
| Female (ref. male) | **1.244 (1.014-1.526)*** | 1.161 (0.941-1.433) | 0.983 (0.822-1.176) | **1.139 (1.014-1.28)*** | 0.91 (0.766-1.082) |
| **AGE CATEGORY** |  |  |  |  |  |
| Year of birth 2000-2001 (ref. 1999 or earlier) | **1.543 (1.259-1.891)***** | **1.394 (1.067-1.822)*** | 0.945 (0.808-1.104) | 1.03 (0.911-1.164) | 0.983 (0.778-1.24) |
| Year of birth 2002 or later (ref. 1999 or earlier) | 2.208 (0.973-5.01) | **1.638 (1.235-2.173)**** | 0.841 (0.407-1.739) | 1.084 (0.949-1.239) | **2.044 (1.231-3.391)**** |
| **INCOME CATEGORY** |  |  |  |  |  |
| Percentile 71-90 (ref. 1-70) | 1.199 (0.918-1.564) | 1.169 (0.887-1.539) | **1.631 (1.198-2.22)**** | **1.243 (1.045-1.478)*** | 0.895 (0.675-1.187) |
| Percentile 91-100 (ref. 1-70) | **1.482 (1.174-1.872)**** | 1.22 (0.96-1.55) | **1.387 (1.058-1.818)*** | 1.154 (0.987-1.35) | 1.019 (0.787-1.319) |
| **ASSETS CATEGORY** |  |  |  |  |  |
| Percentile 41-80 (ref. 1-40) | 1.278 (0.992-1.648) | 1.078 (0.826-1.407) | 1.177 (0.858-1.614) | **1.238 (1.05-1.461)*** | **1.59 (1.182-2.138)**** |
| Percentile 81-100 (ref. 1-40) | **1.35 (1.047-1.741)*** | 1.211 (0.937-1.565) | 1.06 (0.775-1.45) | **1.322 (1.117-1.565)**** | **1.628 (1.218-2.175)**** |
| **NR. OF PARENTS ON WELFARE** |  |  |  |  |  |
| 1 or 2 parents (ref. 0) | 0.808 (0.601-1.086) | 0.81 (0.597-1.098) | 0.766 (0.45-1.304) | 0.783 (0.578-1.061) | 0.665 (0.35-1.263) |
| **NR. OF PARENTS WITH ANOTHER SOCIAL SERVICES INCOME** |  |  |  |  |  |
| 1 or 2 parents (ref. 0) | 0.887 (0.645-1.22) | 0.789 (0.552-1.128) | **1.602 (1.069-2.401)*** | 0.828 (0.677-1.012) | 0.994 (0.723-1.367) |
| **NR. OF PARENTS WITH BIG-REGISTRATION** |  |  |  |  |  |
| 1 or 2 parents (ref. 0) | 0.964 (0.741-1.254) | 0.94 (0.719-1.229) | **1.677 (1.262-2.229)***** | **1.222 (1.04-1.436)*** | 1.009 (0.811-1.256) |
| **NR. OF PARENTS WORKING IN PSVA EDUCATION** |  |  |  |  |  |
| 1 or 2 parents (ref. 0) | 1.246 (0.856-1.813) | 1.021 (0.685-1.522) | 1.206 (0.879-1.653) | 1.049 (0.865-1.272) | 1.05 (0.807-1.366) |
| **MIGRATION BACKGROUND** |  |  |  |  |  |
| TMSDI (ref. no migration background) | **0.698 (0.533-0.914)**** | 0.951 (0.73-1.239) | **0.522 (0.376-0.724)***** | **0.696 (0.581-0.834)***** | **0.517 (0.37-0.723)***** |
| Other (ref. no migration background) | 0.839 (0.677-1.04) | 0.85 (0.681-1.061) | **0.572 (0.489-0.669)***** | 0.935 (0.844-1.036) | **0.663 (0.521-0.844)**** |
| **URBANITY DEGREE** |  |  |  |  |  |
| Average (ref. (very) strongly urban) | 1.147 (0.879-1.498) | 0.767 (0.582-1.009) | 1.171 (0.884-1.551) | **1.189 (1.021-1.385)*** | 0.919 (0.741-1.14) |
| Weakly urban/not urban (ref. (very) strongly urban) | 1.12 (0.887-1.414) | 0.795 (0.622-1.016) | 1.202 (0.949-1.523) | **1.209 (1.059-1.38)**** | 1.06 (0.881-1.274) |

*p<.05; **p<0.01; ***p<0.001

Ref. = reference category; PSVA = Primary, Secondary or Vocational or Agrarian; TMSDI: Turkish, Moroccan, Surinamese, Dutch Caribbean, or Indonesian; Urbanity Degree of postal code of residential address in 2015. Results in **bold** are statistically significant (p<.05).

|  | **SU4_2020** | **SU5_2019** | **SU5_2020** | **SU6_2019** | **SU6_2020** |
| --- | --- | --- | --- | --- | --- |
|  | **Biomedical Sciences; Biomedical Engineering; Clinical Technology** | **Biology; Biotechnology; Nutrition and Health; Veterinary Medicine; Nanobiology** | **Biology; Biotechnology; Nutrition and Health; Veterinary Medicine; Nanobiology** | **Artificial Intelligence; Industrial Design** | **Artificial Intelligence; Industrial Design** |
|  | **OR (95% CI)** | **OR (95% CI)** | **OR (95% CI)** | **OR (95% CI)** | **OR (95% CI)** |
| **GENDER** |  |  |  |  |  |
| Female (ref. male) | 1.043 (0.873-1.247) | **0.52 (0.411-0.659)***** | **0.74 (0.572-0.958)*** | 1.07 (0.84-1.363) | 0.836 (0.634-1.102) |
| **AGE CATEGORY** |  |  |  |  |  |
| Year of birth 2000-2001 (ref. 1999 or earlier) | 0.827 (0.564-1.213) | **2.028 (1.602-2.568)***** | **2.238 (1.558-3.214)***** | 1.049 (0.785-1.402) | 1.136 (0.72-1.794) |
| Year of birth 2002 or later (ref. 1999 or earlier) | 1.098 (0.753-1.6) | **6.11 (2.909-12.834)***** | **2.862 (1.985-4.128)***** | 1.54 (0.666-3.558) | 1.286 (0.808-2.046) |
| **INCOME CATEGORY** |  |  |  |  |  |
| Percentile 71-90 (ref. 1-70) | 0.981 (0.736-1.307) | **1.624 (1.134-2.325)**** | 1.323 (0.883-1.983) | **1.813 (1.176-2.796)**** | 0.927 (0.561-1.534) |
| Percentile 91-100 (ref. 1-70) | 1.253 (0.971-1.619) | **1.552 (1.118-2.154)**** | 1.273 (0.872-1.858) | **1.455 (1.009-2.096)*** | 1.047 (0.665-1.65) |
| **ASSETS CATEGORY** |  |  |  |  |  |
| Percentile 41-80 (ref. 1-40) | **1.603 (1.206-2.132)**** | 1.286 (0.852-1.94) | **1.636 (1.051-2.548)*** | 0.941 (0.581-1.525) | 1.241 (0.74-2.08) |
| Percentile 81-100 (ref. 1-40) | **1.711 (1.293-2.266)***** | **1.503 (1.003-2.252)*** | **1.579 (1.021-2.44)*** | 0.909 (0.57-1.45) | 1.11 (0.675-1.827) |
| **NR. OF PARENTS ON WELFARE** |  |  |  |  |  |
| 1 or 2 parents (ref. 0) | **0.547 (0.308-0.971)*** | 1.079 (0.416-2.8) | 0.848 (0.293-2.46) | 0.764 (0.325-1.795) | 1.286 (0.451-3.67) |
| **NR. OF PARENTS WITH ANOTHER SOCIAL SERVICES INCOME** |  |  |  |  |  |
| 1 or 2 parents (ref. 0) | 1.193 (0.839-1.696) | **0.625 (0.412-0.948)*** | 0.699 (0.433-1.129) | 1.07 (0.614-1.863) | 1.126 (0.59-2.152) |
| **NR. OF PARENTS WITH BIG-REGISTRATION** |  |  |  |  |  |
| 1 or 2 parents (ref. 0) | 0.951 (0.751-1.205) | 0.82 (0.627-1.072) | 0.825 (0.609-1.117) | 0.956 (0.669-1.366) | 0.897 (0.61-1.317) |
| **NR. OF PARENTS WORKING IN PSVA EDUCATION** |  |  |  |  |  |
| 1 or 2 parents (ref. 0) | 1.159 (0.869-1.546) | 0.961 (0.685-1.348) | 0.982 (0.65-1.484) | 1.044 (0.694-1.573) | 1.522 (0.891-2.6) |
| **MIGRATION BACKGROUND** |  |  |  |  |  |
| TMSDI (ref. no migration background) | **0.571 (0.405-0.806)**** | 1.067 (0.632-1.799) | 1.203 (0.651-2.221) | 1.346 (0.762-2.376) | 1.513 (0.771-2.967) |
| Other (ref. no migration background) | **0.763 (0.603-0.965)*** | **1.642 (1.22-2.209)**** | **2.863 (2.099-3.905)***** | 1.233 (0.924-1.644) | 1.175 (0.848-1.628) |
| **URBANITY DEGREE** |  |  |  |  |  |
| Average (ref. (very) strongly urban) | 1.155 (0.923-1.446) | 0.937 (0.699-1.254) | 0.933 (0.659-1.322) | 1.009 (0.71-1.435) | 1.216 (0.817-1.812) |
| Weakly urban/not urban (ref. (very) strongly urban) | **1.266 (1.045-1.533)*** | **0.73 (0.576-0.926)*** | 0.831 (0.636-1.086) | 0.825 (0.62-1.097) | 1.159 (0.827-1.624) |

*p<.05; **p<0.01; ***p<0.001

Ref. = reference category; PSVA = Primary, Secondary or Vocational or Agrarian; TMSDI: Turkish, Moroccan, Surinamese, Dutch Caribbean, or Indonesian; Urbanity Degree of postal code of residential address in 2015. Results in **bold** are statistically significant (p<.05).

|  | **SU7_2019** | **SU7_2020** | **SU8_2019** | **SU8_2020** | **SU9_2019** |
| --- | --- | --- | --- | --- | --- |
|  | **Architecture, Urbanism & Building Sciences; Mechanical Engineering; Aerospace Engineering; Computer Science & Engineering; Global Sustainability Science** | **Architecture, Urbanism & Building Sciences; Mechanical Engineering; Aerospace Engineering; Computer Science & Engineering; Global Sustainability Science** | **Business Administration; International Business; International Business Administration; Tax Law; Industrial Engineering & Management Science** | **Business Administration; International Business; International Business Administration; Tax Law; Industrial Engineering & Management Science** | **International Relations and International Organization; Political Sciences; Criminology** |
|  | **OR (95% CI)** | **OR (95% CI)** | **OR (95% CI)** | **OR (95% CI)** | **OR (95% CI)** |
| **GENDER** |  |  |  |  |  |
| Female (ref. male) | 1.161 (0.985-1.368) | **1.72 (1.461-2.025)***** | **1.24 (1.043-1.474)*** | 1.141 (0.967-1.347) | **0.603 (0.495-0.735)***** |
| **AGE CATEGORY** |  |  |  |  |  |
| Year of birth 2000-2001 (ref. 1999 or earlier) | **1.64 (1.366-1.968)***** | 1.106 (0.84-1.458) | 0.941 (0.76-1.166) | 1.261 (0.927-1.716) | **1.661 (1.38-1.999)***** |
| Year of birth 2002 or later (ref. 1999 or earlier) | **1.999 (1.251-3.194)**** | **1.344 (1.02-1.773)*** | 2.219 (0.871-5.65) | 1.131 (0.83-1.541) | **3.459 (1.586-7.545)**** |
| **INCOME CATEGORY** |  |  |  |  |  |
| Percentile 71-90 (ref. 1-70) | **1.756 (1.332-2.316)***** | **1.331 (1.008-1.757)*** | 1.008 (0.666-1.524) | 1.17 (0.731-1.874) | **1.42 (1.063-1.897)*** |
| Percentile 91-100 (ref. 1-70) | **2.195 (1.721-2.799)***** | **1.922 (1.502-2.459)***** | 1.243 (0.864-1.788) | 0.824 (0.555-1.222) | **1.607 (1.226-2.106)**** |
| **ASSETS CATEGORY** |  |  |  |  |  |
| Percentile 41-80 (ref. 1-40) | **1.808 (1.375-2.377)***** | **1.815 (1.367-2.41)***** | 0.926 (0.602-1.423) | 1.337 (0.9-1.985) | 0.985 (0.738-1.316) |
| Percentile 81-100 (ref. 1-40) | **2.276 (1.745-2.97)***** | **2.135 (1.615-2.821)***** | 1.234 (0.816-1.866) | 1.193 (0.819-1.739) | 1.287 (0.96-1.725) |
| **NR. OF PARENTS ON WELFARE** |  |  |  |  |  |
| 1 or 2 parents (ref. 0) | **0.221 (0.136-0.358)***** | **0.416 (0.253-0.683)**** | 0.467 (0.191-1.14) | **0.332 (0.137-0.805)*** | **0.437 (0.235-0.813)**** |
| **NR. OF PARENTS WITH ANOTHER SOCIAL SERVICES INCOME** |  |  |  |  |  |
| 1 or 2 parents (ref. 0) | 0.865 (0.614-1.218) | 0.793 (0.564-1.116) | 0.864 (0.513-1.455) | 0.878 (0.521-1.478) | **0.336 (0.232-0.487)***** |
| **NR. OF PARENTS WITH BIG-REGISTRATION** |  |  |  |  |  |
| 1 or 2 parents (ref. 0) | **1.445 (1.108-1.884)**** | 1.279 (0.977-1.675) | 1.162 (0.794-1.7) | 0.976 (0.678-1.406) | 0.825 (0.631-1.079) |
| **NR. OF PARENTS WORKING IN PSVA EDUCATION** |  |  |  |  |  |
| 1 or 2 parents (ref. 0) | 1.006 (0.747-1.355) | **1.379 (1.01-1.883)*** | **2.411 (1.294-4.492)**** | 1.232 (0.785-1.933) | **0.636 (0.474-0.854)**** |
| **MIGRATION BACKGROUND** |  |  |  |  |  |
| TMSDI (ref. no migration background) | **0.325 (0.25-0.422)***** | **0.552 (0.424-0.718)***** | **0.39 (0.28-0.543)***** | **0.535 (0.379-0.756)***** | 0.736 (0.521-1.041) |
| Other (ref. no migration background) | **0.711 (0.609-0.831)***** | **0.791 (0.68-0.92)**** | **0.689 (0.571-0.831)***** | 1.007 (0.843-1.202) | **3.5 (2.815-4.351)***** |
| **URBANITY DEGREE** |  |  |  |  |  |
| Average (ref. (very) strongly urban) | **1.311 (1.039-1.654)*** | 1.247 (0.979-1.587) | 1.299 (0.93-1.813) | 1.096 (0.796-1.508) | 0.998 (0.784-1.27) |
| Weakly urban/not urban (ref. (very) strongly urban) | **1.522 (1.228-1.885)***** | 1.163 (0.94-1.438) | **1.977 (1.472-2.655)***** | **1.605 (1.199-2.148)**** | 1.226 (0.992-1.516) |

*p<.05; **p<0.01; ***p<0.001; PSVA = Primary, Secondary or Vocational or Agrarian;

Ref. = reference category; PSVA = Primary, Secondary or Vocational or Agrarian; TMSDI: Turkish, Moroccan, Surinamese, Dutch Caribbean, or Indonesian; Urbanity Degree of postal code of residential address in 2015. Results in **bold** are statistically significant (p<.05).

|  | **SU9_2020** |
| --- | --- |
|  | **International Relations and International Organization; Political Sciences; Criminology** |
|  | **OR (95% CI)** |
| **GENDER** |  |
| Female (ref. male) | **0.586 (0.488-0.704)***** |
| **AGE CATEGORY** |  |
| Year of birth 2000-2001 (ref. 1999 or earlier) | **1.856 (1.472-2.34)***** |
| Year of birth 2002 or later (ref. 1999 or earlier) | **2.119 (1.66-2.705)***** |
| **INCOME CATEGORY** |  |
| Percentile 71-90 (ref. 1-70) | **1.347 (1.023-1.773)*** |
| Percentile 91-100 (ref. 1-70) | **1.724 (1.336-2.223)***** |
| **ASSETS CATEGORY** |  |
| Percentile 41-80 (ref. 1-40) | **1.662 (1.284-2.15)***** |
| Percentile 81-100 (ref. 1-40) | **1.967 (1.512-2.56)***** |
| **NR. OF PARENTS ON WELFARE** |  |
| 1 or 2 parents (ref. 0) | 0.612 (0.353-1.061) |
| **NR. OF PARENTS WITH ANOTHER SOCIAL SERVICES INCOME** |  |
| 1 or 2 parents (ref. 0) | **0.409 (0.293-0.571)***** |
| **NR. OF PARENTS WITH BIG-REGISTRATION** |  |
| 1 or 2 parents (ref. 0) | **0.76 (0.587-0.985)*** |
| **NR. OF PARENTS WORKING IN PSVA EDUCATION** |  |
| 1 or 2 parents (ref. 0) | 1.018 (0.771-1.345) |
| **MIGRATION BACKGROUND** |  |
| TMSDI (ref. no migration background) | 0.751 (0.548-1.027) |
| Other (ref. no migration background) | **3.329 (2.736-4.051)***** |
| **URBANITY DEGREE** |  |
| Average (ref. (very) strongly urban) | 0.889 (0.709-1.117) |
| Weakly urban/not urban (ref. (very) strongly urban) | 0.976 (0.802-1.186) |

*p<.05; **p<0.01; ***p<0.001

Ref. = reference category; PSVA = Primary, Secondary or Vocational or Agrarian; TMSDI: Turkish, Moroccan, Surinamese, Dutch Caribbean, or Indonesian; Urbanity Degree of postal code of residential address in 2015. Results in **bold** are statistically significant (p<.05).
