# Supplementary material for: Inequality of opportunity in selection procedures limits diversity in higher education: An intersectional study of Dutch selective higher education programs
Source: PLoS One. 2023 Oct 13;18(10):e0292805. doi: 10.1371/journal.pone.0292805 (PMC10575509; doi:10.1371/journal.pone.0292805)
Supplement: S4 Table — *p < .05; **p<0.01; ***p<0.001. Ref. = reference category; PSVA = Primary, Secondary or Vocational or Agrarian; TMSDI: Turkish, Moroccan, Surinamese, Dutch Caribbean, or Indonesian; Urbanity Degree of postal code of residential address in 2015. Results in bold are statistically significant (p < .05). (DOCX) [file pone.0292805.s004.docx]

# **S4: Results of multivariable logistic regression for the final model for each cluster, performed on applicants eligible for placement**

|  | **TOTAL DATASET** | **SH1_2019** | **SH1_2020** | **SH2_2019** | **SH2_2020** |
| --- | --- | --- | --- | --- | --- |
|  |  | **Allied Medical Care; Nursing; Midwifery** | **Allied Medical Care; Nursing; Midwifery** | **Dental Hygiene. Denturism. Optometry** | **Dental Hygiene. Denturism. Optometry** |
|  | **OR (95% CI)** | **OR (95% CI)** | **OR (95% CI)** | **OR (95% CI)** | **OR (95% CI)** |
| **SEX** |  |  |  |  |  |
| Female (ref. male) | **0.873 (0.844-0.902)***** | **2.022 (1.535-2.662)***** | **1.954 (1.496-2.552)***** | 0.82 (0.594-1.133) | 0.795 (0.566-1.115) |
| **AGE CATEGORY** |  |  |  |  |  |
| Year of birth 2000-2001 (ref. 1999 or earlier) | **1.052 (1.011-1.094)*** | **0.771 (0.62-0.959)*** | 0.787 (0.599-1.035) | 1.265 (0.99-1.616) | 0.821 (0.618-1.091) |
| Year of birth 2002 or later (ref. 1999 or earlier) | **1.05 (1.002-1.1)*** | **0.468 (0.349-0.627)***** | **0.537 (0.413-0.698)***** | 0.81 (0.527-1.246) | 0.862 (0.629-1.181) |
| **INCOME CATEGORY** |  |  |  |  |  |
| Percentile 71-90 (ref. 1-70) | **1.118 (1.063-1.177)***** | 0.753 (0.555-1.022) | **1.36 (1.004-1.841)*** | 1.228 (0.896-1.684) | 0.903 (0.651-1.254) |
| Percentile 91-100 (ref. 1-70) | **1.128 (1.073-1.186)***** | **0.598 (0.438-0.816)**** | 1.019 (0.749-1.386) | 1.308 (0.906-1.89) | 0.917 (0.631-1.333) |
| **ASSETS CATEGORY** |  |  |  |  |  |
| Percentile 41-80 (ref. 1-40) | **1.187 (1.128-1.248)***** | 1.322 (0.979-1.785) | 1.211 (0.909-1.614) | 1.122 (0.811-1.553) | **1.79 (1.282-2.498)**** |
| Percentile 81-100 (ref. 1-40) | **1.279 (1.213-1.348)***** | 1.233 (0.889-1.709) | 1.249 (0.91-1.714) | **1.512 (1.014-2.255)*** | **1.809 (1.193-2.744)**** |
| **NR. OF PARENTS ON WELFARE** |  |  |  |  |  |
| 1 or 2 parents (ref. 0) | **0.787 (0.721-0.86)***** | 0.86 (0.456-1.624) | 1.393 (0.752-2.581) | 0.892 (0.591-1.347) | 0.818 (0.535-1.252) |
| **NR. OF PARENTS WITH ANOTHER SOCIAL SERVICES INCOME (excl. welfare)** |  |  |  |  |  |
| 1 or 2 parents (ref. 0) | 0.952 (0.898-1.009) | 0.87 (0.6-1.261) | 1.281 (0.873-1.879) | 1.278 (0.888-1.839) | 0.939 (0.63-1.401) |
| **NR. OF PARENTS WITH BIG-REGISTRATION** |  |  |  |  |  |
| 1 or 2 parents (ref. 0) | 1.003 (0.96-1.048) | **1.793 (1.348-2.385)***** | **1.359 (1.026-1.8)*** | **1.677 (1.048-2.683)*** | **1.729 (1.07-2.792)*** |
| **NR. OF PARENTS WORKING IN PSVA EDUCATION** |  |  |  |  |  |
| 1 or 2 parents (ref. 0) | **1.099 (1.04-1.161)**** | **1.459 (1.014-2.098)*** | 1.22 (0.862-1.727) | 0.799 (0.481-1.324) | 0.73 (0.4-1.335) |
| **MIGRATION BACKGROUND** |  |  |  |  |  |
| TMSDI (ref. no migration background) | **0.554 (0.522-0.587)***** | **0.296 (0.189-0.465)***** | **0.436 (0.282-0.673)***** | **0.478 (0.334-0.684)***** | **0.387 (0.261-0.572)***** |
| Other (ref. no migration background) | **0.754 (0.721-0.79)***** | **0.584 (0.402-0.848)**** | **0.581 (0.404-0.835)**** | **0.586 (0.416-0.824)**** | **0.534 (0.368-0.773)**** |
| **URBANITY DEGREE** |  |  |  |  |  |
| Average (ref. (very) strongly urban) | 1.027 (0.985-1.072) | **1.771 (1.345-2.331)***** | **1.453 (1.1-1.918)**** | **1.377 (1.001-1.894)*** | 0.85 (0.614-1.177) |
| Weakly urban/not urban (ref. (very) strongly urban) | 1.03 (0.993-1.069) | **2.505 (1.991-3.153)***** | **2.386 (1.889-3.013)***** | 0.948 (0.698-1.289) | 0.752 (0.548-1.032) |

*p<.05; **p<0.01; ***p<0.001

Ref. = reference category; PSVA = Primary, Secondary or Vocational or Agrarian; TMSDI: Turkish, Moroccan, Surinamese, Dutch Caribbean, or Indonesian; Urbanity Degree of postal code of residential address in 2015. Results in **bold** are statistically significant (p<.05).

|  | **SH3_2019** | **SH3_2020** | **SH4_2019** | **SH4_2020** | **SH5_2019** |
| --- | --- | --- | --- | --- | --- |
|  | **Biology and Medical Laboratory Research; Forensic Science; Medical Imaging and Radiation Therapy** | **Biology and Medical Laboratory Research; Forensic Science; Medical Imaging and Radiation Therapy** | **Physiotherapy; Psychomotoric Therapy/ Psychomotricity; Sport Studies** | **Physiotherapy; Psychomotoric Therapy/ Psychomotricity; Sport Studies** | **Creative Media and Game Technologies; Fashion & Textile Technologies; Industrial Design Engineering; Art and Economics** |
|  | **OR (95% CI)** | **OR (95% CI)** | **OR (95% CI)** | **OR (95% CI)** | **OR (95% CI)** |
| **SEX** |  |  |  |  |  |
| Female (ref. male) | 0.898 (0.661-1.221) | 0.909 (0.666-1.239) | **1.226 (1.011-1.487)*** | **1.614 (1.349-1.932)***** | **2.608 (2.024-3.36)***** |
| **AGE CATEGORY** |  |  |  |  |  |
| Year of birth 2000-2001 (ref. 1999 or earlier) | 1.031 (0.729-1.458) | 1.157 (0.714-1.873) | 0.99 (0.799-1.227) | 0.799 (0.614-1.04) | 0.819 (0.625-1.072) |
| Year of birth 2002 or later (ref. 1999 or earlier) | 0.737 (0.499-1.086) | 0.873 (0.555-1.374) | 0.914 (0.694-1.202) | **0.759 (0.587-0.98)*** | 0.674 (0.446-1.018) |
| **INCOME CATEGORY** |  |  |  |  |  |
| Percentile 71-90 (ref. 1-70) | 1.016 (0.68-1.519) | 0.849 (0.553-1.304) | **1.631 (1.236-2.152)**** | 1.156 (0.882-1.515) | 0.793 (0.542-1.16) |
| Percentile 91-100 (ref. 1-70) | 1.048 (0.689-1.595) | 0.927 (0.597-1.439) | **1.707 (1.294-2.252)***** | 1.105 (0.846-1.445) | 0.996 (0.68-1.46) |
| **ASSETS CATEGORY** |  |  |  |  |  |
| Percentile 41-80 (ref. 1-40) | 1.075 (0.724-1.598) | 0.817 (0.545-1.225) | 0.927 (0.683-1.258) | 1.089 (0.817-1.452) | **1.437 (1.004-2.055)*** |
| Percentile 81-100 (ref. 1-40) | 0.865 (0.557-1.344) | 0.691 (0.437-1.093) | 1.176 (0.848-1.63) | 1.097 (0.81-1.486) | **1.671 (1.134-2.462)**** |
| **NR. OF PARENTS ON WELFARE** |  |  |  |  |  |
| 1 or 2 parents (ref. 0) | 0.94 (0.459-1.928) | 0.855 (0.381-1.918) | 1.335 (0.662-2.69) | 1.195 (0.546-2.615) | 1.421 (0.623-3.242) |
| **NR. OF PARENTS WITH ANOTHER SOCIAL SERVICES INCOME** |  |  |  |  |  |
| 1 or 2 parents (ref. 0) | 1.22 (0.764-1.949) | 1.147 (0.685-1.921) | 0.764 (0.529-1.104) | 0.733 (0.519-1.035) | 1.47 (0.921-2.347) |
| **NR. OF PARENTS WITH BIG-REGISTRATION** |  |  |  |  |  |
| 1 or 2 parents (ref. 0) | 0.921 (0.609-1.391) | 0.966 (0.647-1.443) | 1.053 (0.809-1.37) | **1.441 (1.109-1.872)**** | 1.328 (0.858-2.056) |
| **NR. OF PARENTS WORKING IN PSVA EDUCATION** |  |  |  |  |  |
| 1 or 2 parents (ref. 0) | 1.057 (0.647-1.726) | 1.284 (0.79-2.085) | 0.924 (0.684-1.248) | 0.998 (0.761-1.308) | 1.314 (0.851-2.029) |
| **MIGRATION BACKGROUND** |  |  |  |  |  |
| TMSDI (ref. no migration background) | 1.104 (0.676-1.804) | **1.9 (1.078-3.347)*** | 1.103 (0.663-1.835) | 1.029 (0.637-1.661) | 0.808 (0.505-1.294) |
| Other (ref. no migration background) | 1.285 (0.817-2.019) | 1.425 (0.904-2.247) | 0.875 (0.609-1.258) | 0.757 (0.545-1.053) | 0.814 (0.575-1.152) |
| **URBANITY DEGREE** |  |  |  |  |  |
| Average (ref. (very) strongly urban) | **1.714 (1.186-2.476)**** | **1.477 (1.022-2.133)*** | 0.8 (0.616-1.038) | 0.979 (0.764-1.255) | 0.925 (0.666-1.285) |
| Weakly urban/not urban (ref. (very) strongly urban) | **2.28 (1.664-3.124)***** | **1.856 (1.352-2.547)***** | **0.657 (0.527-0.819)***** | **0.691 (0.565-0.846)***** | 1.083 (0.8-1.467) |

*p<.05; **p<0.01; ***p<0.001

Ref. = reference category; PSVA = Primary, Secondary or Vocational or Agrarian; TMSDI: Turkish, Moroccan, Surinamese, Dutch Caribbean, or Indonesian; Urbanity Degree of postal code of residential address in 2015. Results in **bold** are statistically significant (p<.05).

|  | **SH5_2020** | **SH6_2019** | **SH6_2020** | **SU1_2019** | **SU1_2020** |
| --- | --- | --- | --- | --- | --- |
|  | **Creative Media and Game Technologies; Fashion & Textile Technologies; Industrial Design Engineering; Art and Economics** | **Applied Psychology; Applied Biology; Skin Therapy** | **Applied Psychology; Applied Biology; Skin Therapy** | **Medicine** | **Medicine** |
|  | **OR (95% CI)** | **OR (95% CI)** | **OR (95% CI)** | **OR (95% CI)** | **OR (95% CI)** |
| **SEX** |  |  |  |  |  |
| Female (ref. male) | **1.554 (1.194-2.021)**** | **0.497 (0.406-0.608)***** | **0.798 (0.663-0.96)*** | **1.18 (1.056-1.319)**** | 1.012 (0.908-1.128) |
| **AGE CATEGORY** |  |  |  |  |  |
| Year of birth 2000-2001 (ref. 1999 or earlier) | **1.392 (1.001-1.936)*** | 1.076 (0.911-1.271) | 0.96 (0.797-1.156) | 1.114 (0.988-1.256) | 0.914 (0.778-1.074) |
| Year of birth 2002 or later (ref. 1999 or earlier) | 0.913 (0.643-1.296) | **1.376 (1.07-1.771)*** | 1.164 (0.959-1.413) | **1.889 (1.318-2.709)**** | 1.117 (0.948-1.317) |
| **INCOME CATEGORY** |  |  |  |  |  |
| Percentile 71-90 (ref. 1-70) | 0.773 (0.523-1.144) | 1.214 (0.978-1.508) | 1.169 (0.946-1.445) | 1.028 (0.853-1.24) | 1.137 (0.948-1.364) |
| Percentile 91-100 (ref. 1-70) | 0.806 (0.543-1.197) | 1.217 (0.969-1.528) | 1.068 (0.856-1.332) | 1.142 (0.954-1.367) | 1.183 (0.993-1.411) |
| **ASSETS CATEGORY** |  |  |  |  |  |
| Percentile 41-80 (ref. 1-40) | 1.362 (0.936-1.983) | **1.242 (1.003-1.537)*** | 1.131 (0.918-1.393) | 0.919 (0.772-1.094) | **1.274 (1.072-1.514)**** |
| Percentile 81-100 (ref. 1-40) | 1.433 (0.95-2.161) | **1.583 (1.247-2.011)***** | **1.307 (1.031-1.658)*** | 1.167 (0.977-1.396) | **1.389 (1.161-1.662)***** |
| **NR. OF PARENTS ON WELFARE** |  |  |  |  |  |
| 1 or 2 parents (ref. 0) | 0.623 (0.296-1.315) | 0.863 (0.62-1.2) | 0.806 (0.581-1.118) | 0.872 (0.643-1.182) | 0.873 (0.652-1.17) |
| **NR. OF PARENTS WITH ANOTHER SOCIAL SERVICES INCOME** |  |  |  |  |  |
| 1 or 2 parents (ref. 0) | 1.208 (0.744-1.962) | 0.848 (0.661-1.086) | 1.256 (0.986-1.6) | 0.916 (0.753-1.113) | 0.974 (0.804-1.181) |
| **NR. OF PARENTS WITH BIG-REGISTRATION** |  |  |  |  |  |
| 1 or 2 parents (ref. 0) | 1.636 (0.983-2.724) | 1.164 (0.905-1.498) | 1.146 (0.906-1.45) | 1.135 (0.999-1.289) | 1.088 (0.959-1.236) |
| **NR. OF PARENTS WORKING IN PSVA EDUCATION** |  |  |  |  |  |
| 1 or 2 parents (ref. 0) | 1.1 (0.705-1.715) | **1.454 (1.054-2.007)*** | 0.997 (0.745-1.333) | 1.006 (0.839-1.206) | 1.044 (0.873-1.249) |
| **MIGRATION BACKGROUND** |  |  |  |  |  |
| TMSDI (ref. no migration background) | **0.542 (0.344-0.854)**** | **0.699 (0.543-0.899)**** | **0.585 (0.46-0.746)***** | **0.673 (0.552-0.82)***** | 0.841 (0.697-1.016) |
| Other (ref. no migration background) | 0.807 (0.563-1.158) | 0.844 (0.667-1.068) | **0.796 (0.637-0.993)*** | 0.982 (0.844-1.142) | 1.052 (0.911-1.215) |
| **URBANITY DEGREE** |  |  |  |  |  |
| Average (ref. (very) strongly urban) | 0.817 (0.578-1.155) | 1.215 (0.979-1.507) | 1.113 (0.91-1.361) | 0.974 (0.847-1.121) | 0.981 (0.856-1.125) |
| Weakly urban/not urban (ref. (very) strongly urban) | **0.71 (0.519-0.97)*** | 1.024 (0.854-1.228) | 1.151 (0.961-1.377) | 0.927 (0.821-1.046) | 1.054 (0.935-1.188) |

*p<.05; **p<0.01; ***p<0.001

Ref. = reference category; PSVA = Primary, Secondary or Vocational or Agrarian; TMSDI: Turkish, Moroccan, Surinamese, Dutch Caribbean, or Indonesian; Urbanity Degree of postal code of residential address in 2015. Results in **bold** are statistically significant (p<.05).

|  | **SU2_2019** | **SU2_2020** | **SU3_2019** | **SU3_2020** | **SU4_2019** |
| --- | --- | --- | --- | --- | --- |
|  | **Dentistry; Pharmacy** | **Dentistry; Pharmacy** | **Psychobiology; Psychology** | **Psychobiology; Psychology** | **Biomedical Sciences; Biomedical Engineering; Clinical Technology** |
|  | **OR (95% CI)** | **OR (95% CI)** | **OR (95% CI)** | **OR (95% CI)** | **OR (95% CI)** |
| **SEX** |  |  |  |  |  |
| Female (ref. male) | 1.179 (0.945-1.472) | 1.148 (0.914-1.443) | 1.024 (0.788-1.33) | 1.141 (0.989-1.316) | 0.905 (0.757-1.081) |
| **AGE CATEGORY** |  |  |  |  |  |
| Year of birth 2000-2001 (ref. 1999 or earlier) | **1.316 (1.047-1.654)*** | 1.303 (0.956-1.775) | 1.003 (0.79-1.273) | 0.982 (0.837-1.152) | 0.93 (0.726-1.192) |
| Year of birth 2002 or later (ref. 1999 or earlier) | 1.908 (0.783-4.649) | **1.498 (1.079-2.08)*** | 1.605 (0.482-5.34) | 1.099 (0.927-1.303) | **1.794 (1.069-3.011)*** |
| **INCOME CATEGORY** |  |  |  |  |  |
| Percentile 71-90 (ref. 1-70) | 1.109 (0.793-1.55) | 1.098 (0.773-1.56) | **1.535 (1.089-2.165)*** | 1.103 (0.91-1.338) | **0.727 (0.529-0.998)*** |
| Percentile 91-100 (ref. 1-70) | 1.368 (0.978-1.916) | 1.147 (0.803-1.639) | 1.363 (0.985-1.885) | 0.964 (0.801-1.16) | 0.803 (0.593-1.088) |
| **ASSETS CATEGORY** |  |  |  |  |  |
| Percentile 41-80 (ref. 1-40) | 1.081 (0.781-1.498) | 0.905 (0.639-1.281) | 0.884 (0.614-1.273) | 1.052 (0.871-1.271) | **1.449 (1.048-2.004)*** |
| Percentile 81-100 (ref. 1-40) | 1.099 (0.773-1.562) | 0.981 (0.674-1.428) | 0.785 (0.541-1.14) | 1.125 (0.923-1.37) | **1.436 (1.034-1.994)*** |
| **NR. OF PARENTS ON WELFARE** |  |  |  |  |  |
| 1 or 2 parents (ref. 0) | 1.036 (0.675-1.59) | 0.982 (0.625-1.543) | 0.723 (0.39-1.34) | 0.952 (0.67-1.354) | 1.019 (0.478-2.171) |
| **NR. OF PARENTS WITH ANOTHER SOCIAL SERVICES INCOME** |  |  |  |  |  |
| 1 or 2 parents (ref. 0) | 1.105 (0.786-1.555) | 0.701 (0.473-1.039) | 1.425 (0.923-2.199) | 0.886 (0.716-1.097) | 0.984 (0.706-1.372) |
| **NR. OF PARENTS WITH BIG-REGISTRATION** |  |  |  |  |  |
| 1 or 2 parents (ref. 0) | 0.842 (0.629-1.126) | 0.892 (0.661-1.203) | 1.272 (0.936-1.727) | **1.203 (1.015-1.426)*** | 0.96 (0.765-1.205) |
| **NR. OF PARENTS WORKING IN PSVA EDUCATION** |  |  |  |  |  |
| 1 or 2 parents (ref. 0) | 1.161 (0.786-1.714) | 0.972 (0.642-1.474) | 0.95 (0.674-1.338) | 1.02 (0.836-1.244) | 1.024 (0.782-1.341) |
| **MIGRATION BACKGROUND** |  |  |  |  |  |
| TMSDI (ref. no migration background) | 0.782 (0.552-1.106) | 0.948 (0.677-1.328) | 0.775 (0.47-1.276) | **0.71 (0.568-0.888)**** | **0.562 (0.381-0.828)**** |
| Other (ref. no migration background) | 1.069 (0.801-1.425) | 0.77 (0.572-1.037) | **0.727 (0.538-0.982)*** | **0.787 (0.661-0.936)**** | **0.666 (0.505-0.879)**** |
| **URBANITY DEGREE** |  |  |  |  |  |
| Average (ref. (very) strongly urban) | 1.047 (0.789-1.39) | **0.717 (0.535-0.961)*** | 1.071 (0.801-1.432) | 1.141 (0.974-1.336) | 0.877 (0.702-1.096) |
| Weakly urban/not urban (ref. (very) strongly urban) | 0.973 (0.752-1.26) | **0.719 (0.548-0.942)*** | 1.072 (0.838-1.371) | 1.115 (0.971-1.281) | 0.951 (0.783-1.156) |

*p<.05; **p<0.01; ***p<0.001

Ref. = reference category; PSVA = Primary, Secondary or Vocational or Agrarian; TMSDI: Turkish, Moroccan, Surinamese, Dutch Caribbean, or Indonesian; Urbanity Degree of postal code of residential address in 2015. Results in **bold** are statistically significant (p<.05).

|  | **SU4_2020** | **SU5_2019** | **SU5_2020** | **SU6_2019** | **SU6_2020** |
| --- | --- | --- | --- | --- | --- |
|  | **Biomedical Sciences; Biomedical Engineering; Clinical Technology** | **Biology; Biotechnology; Nutrition and Health; Veterinary Medicine; Nanobiology** | **Biology; Biotechnology; Nutrition and Health; Veterinary Medicine; Nanobiology** | **Artificial Intelligence; Industrial Design** | **Artificial Intelligence; Industrial Design** |
|  | **OR (95% CI)** | **OR (95% CI)** | **OR (95% CI)** | **OR (95% CI)** | **OR (95% CI)** |
| **SEX** |  |  |  |  |  |
| Female (ref. male) | 1.017 (0.844-1.224) | **0.562 (0.436-0.724)***** | **0.736 (0.557-0.974)*** | 1.104 (0.848-1.437) | 0.817 (0.608-1.097) |
| **AGE CATEGORY** |  |  |  |  |  |
| Year of birth 2000-2001 (ref. 1999 or earlier) | 0.735 (0.48-1.123) | **1.926 (1.484-2.501)***** | **1.872 (1.256-2.79)**** | 0.895 (0.639-1.253) | 1.295 (0.792-2.118) |
| Year of birth 2002 or later (ref. 1999 or earlier) | 0.962 (0.631-1.466) | **5.729 (2.668-12.303)***** | **2.455 (1.639-3.676)***** | 1.123 (0.47-2.683) | 1.46 (0.88-2.421) |
| **INCOME CATEGORY** |  |  |  |  |  |
| Percentile 71-90 (ref. 1-70) | 0.772 (0.556-1.072) | **1.547 (1.044-2.29)*** | 1.267 (0.81-1.983) | **1.866 (1.172-2.972)**** | 0.855 (0.478-1.532) |
| Percentile 91-100 (ref. 1-70) | 0.983 (0.722-1.337) | 1.365 (0.944-1.975) | 1.189 (0.771-1.833) | **1.523 (1.008-2.301)*** | 1.049 (0.601-1.829) |
| **ASSETS CATEGORY** |  |  |  |  |  |
| Percentile 41-80 (ref. 1-40) | **1.49 (1.083-2.049)*** | 1.098 (0.699-1.724) | 1.519 (0.931-2.48) | 0.889 (0.518-1.526) | 1.173 (0.639-2.151) |
| Percentile 81-100 (ref. 1-40) | **1.495 (1.082-2.066)*** | 1.348 (0.859-2.117) | 1.436 (0.881-2.34) | 0.878 (0.513-1.502) | 1.102 (0.602-2.018) |
| **NR. OF PARENTS ON WELFARE** |  |  |  |  |  |
| 1 or 2 parents (ref. 0) | 0.962 (0.478-1.939) | 1.955 (0.626-6.104) | 1.569 (0.468-5.265) | 0.97 (0.346-2.719) | 2.113 (0.555-8.048) |
| **NR. OF PARENTS WITH ANOTHER SOCIAL SERVICES INCOME** |  |  |  |  |  |
| 1 or 2 parents (ref. 0) | 1.344 (0.923-1.958) | 0.752 (0.482-1.173) | 0.963 (0.575-1.613) | 1.108 (0.625-1.963) | 1.399 (0.698-2.806) |
| **NR. OF PARENTS WITH BIG-REGISTRATION** |  |  |  |  |  |
| 1 or 2 parents (ref. 0) | 0.877 (0.688-1.118) | 0.846 (0.634-1.129) | 0.982 (0.714-1.351) | 1.046 (0.72-1.519) | 0.998 (0.673-1.481) |
| **NR. OF PARENTS WORKING IN PSVA EDUCATION** |  |  |  |  |  |
| 1 or 2 parents (ref. 0) | 1.131 (0.84-1.524) | 0.924 (0.646-1.321) | 1.052 (0.684-1.618) | 1.082 (0.707-1.655) | 1.651 (0.958-2.847) |
| **MIGRATION BACKGROUND** |  |  |  |  |  |
| TMSDI (ref. no migration background) | **0.615 (0.414-0.913)*** | 1.264 (0.651-2.452) | 0.794 (0.34-1.854) | 1.283 (0.657-2.503) | 1.514 (0.685-3.349) |
| Other (ref. no migration background) | 0.845 (0.649-1.099) | 1.2 (0.813-1.772) | **1.546 (1.016-2.353)*** | 1.039 (0.713-1.515) | 0.828 (0.555-1.235) |
| **URBANITY DEGREE** |  |  |  |  |  |
| Average (ref. (very) strongly urban) | 1.12 (0.889-1.41) | 0.953 (0.702-1.295) | 0.909 (0.632-1.307) | 1.006 (0.701-1.443) | 1.312 (0.868-1.984) |
| Weakly urban/not urban (ref. (very) strongly urban) | 1.204 (0.985-1.472) | **0.724 (0.562-0.933)*** | 0.85 (0.64-1.128) | 0.823 (0.611-1.111) | 1.245 (0.876-1.771) |

*p<.05; **p<0.01; ***p<0.001

Ref. = reference category; PSVA = Primary, Secondary or Vocational or Agrarian; TMSDI: Turkish, Moroccan, Surinamese, Dutch Caribbean, or Indonesian; Urbanity Degree of postal code of residential address in 2015. Results in **bold** are statistically significant (p<.05).

|  | **SU7_2019** | **SU7_2020** | **SU8_2019** | **SU8_2020** | **SU9_2019** |
| --- | --- | --- | --- | --- | --- |
|  | **Architecture. Urbanism & Building Sciences; Mechanical Engineering; Aerospace Engineering; Computer Science & Engineering; Global Sustainability Science** | **Architecture. Urbanism & Building Sciences; Mechanical Engineering; Aerospace Engineering; Computer Science & Engineering; Global Sustainability Science** | **Business Administration; International Business; International Business Administration; Tax Law; Industrial Engineering & Management Science** | **Business Administration; International Business; International Business Administration; Tax Law; Industrial Engineering & Management Science** | **International Relations and International Organization; Political Sciences; Criminology** |
|  | **OR (95% CI)** | **OR (95% CI)** | **OR (95% CI)** | **OR (95% CI)** | **OR (95% CI)** |
| **SEX** |  |  |  |  |  |
| Female (ref. male) | 1.146 (0.929-1.413) | **1.573 (1.283-1.929)***** | 0.927 (0.711-1.208) | 1.008 (0.777-1.308) | **0.637 (0.506-0.802)***** |
| **AGE CATEGORY** |  |  |  |  |  |
| Year of birth 2000-2001 (ref. 1999 or earlier) | **1.575 (1.237-2.005)***** | 0.961 (0.665-1.389) | 0.954 (0.644-1.411) | 0.86 (0.457-1.62) | **1.837 (1.474-2.29)***** |
| Year of birth 2002 or later (ref. 1999 or earlier) | **2.207 (1.206-4.039)*** | 1.41 (0.976-2.036) | 2.386 (0.532-10.706) | 0.916 (0.487-1.724) | **4.852 (2.163-10.884)***** |
| **INCOME CATEGORY** |  |  |  |  |  |
| Percentile 71-90 (ref. 1-70) | 1.167 (0.843-1.616) | 0.939 (0.678-1.3) | 0.771 (0.479-1.242) | 0.858 (0.496-1.485) | 1.187 (0.851-1.656) |
| Percentile 91-100 (ref. 1-70) | 1.318 (0.975-1.781) | 1.195 (0.875-1.632) | 0.892 (0.571-1.393) | **0.586 (0.358-0.959)*** | 1.285 (0.932-1.772) |
| **ASSETS CATEGORY** |  |  |  |  |  |
| Percentile 41-80 (ref. 1-40) | 1.032 (0.739-1.441) | 1.353 (0.958-1.912) | 0.616 (0.367-1.034) | 1.05 (0.651-1.693) | 0.768 (0.555-1.062) |
| Percentile 81-100 (ref. 1-40) | 1.161 (0.832-1.62) | **1.442 (1.014-2.052)*** | 0.782 (0.467-1.31) | 0.964 (0.6-1.548) | 0.964 (0.69-1.346) |
| **NR. OF PARENTS ON WELFARE** |  |  |  |  |  |
| 1 or 2 parents (ref. 0) | **0.419 (0.219-0.8)**** | 0.627 (0.313-1.257) | 0.72 (0.245-2.116) | 0.405 (0.142-1.149) | 0.803 (0.392-1.645) |
| **NR. OF PARENTS WITH ANOTHER SOCIAL SERVICES INCOME** |  |  |  |  |  |
| 1 or 2 parents (ref. 0) | 1.146 (0.778-1.688) | 0.976 (0.671-1.42) | 0.899 (0.509-1.586) | 0.922 (0.527-1.612) | **0.576 (0.385-0.861)**** |
| **NR. OF PARENTS WITH BIG-REGISTRATION** |  |  |  |  |  |
| 1 or 2 parents (ref. 0) | 1.297 (0.973-1.729) | 1.193 (0.896-1.59) | 1.073 (0.706-1.631) | 1.044 (0.703-1.551) | 1.14 (0.86-1.513) |
| **NR. OF PARENTS WORKING IN PSVA EDUCATION** |  |  |  |  |  |
| 1 or 2 parents (ref. 0) | 0.838 (0.613-1.146) | 1.264 (0.91-1.755) | **2.751 (1.367-5.534)**** | 1.443 (0.876-2.376) | 0.792 (0.578-1.083) |
| **MIGRATION BACKGROUND** |  |  |  |  |  |
| TMSDI (ref. no migration background) | **0.326 (0.224-0.474)***** | 0.751 (0.497-1.135) | **0.32 (0.197-0.519)***** | **0.517 (0.304-0.881)*** | 0.795 (0.516-1.224) |
| Other (ref. no migration background) | **0.701 (0.544-0.905)**** | **0.704 (0.56-0.886)**** | **0.424 (0.306-0.588)***** | 0.725 (0.524-1.003) | 1.072 (0.788-1.459) |
| **URBANITY DEGREE** |  |  |  |  |  |
| Average (ref. (very) strongly urban) | 1.17 (0.917-1.494) | 1.123 (0.871-1.448) | 1.086 (0.764-1.543) | 1.056 (0.756-1.476) | 0.944 (0.732-1.216) |
| Weakly urban/not urban (ref. (very) strongly urban) | 1.24 (0.987-1.556) | 1.01 (0.806-1.267) | **1.559 (1.139-2.132)**** | **1.479 (1.088-2.009)*** | 1.156 (0.924-1.446) |

p<.05; **p<0.01; ***p<0.001; PSVA = Primary, Secondary or Vocational or Agrarian;

Ref. = reference category; PSVA = Primary, Secondary or Vocational or Agrarian; TMSDI: Turkish, Moroccan, Surinamese, Dutch Caribbean, or Indonesian; Urbanity Degree of postal code of residential address in 2015. Results in **bold** are statistically significant (p<.05).

|  | **SU9_2020** |
| --- | --- |
|  | **International Relations and International Organization; Political Sciences; Criminology** |
|  | **OR (95% CI)** |
| **SEX** |  |
| Female (ref. male) | **0.648 (0.524-0.801)***** |
| **AGE CATEGORY** |  |
| Year of birth 2000-2001 (ref. 1999 or earlier) | **1.561 (1.192-2.044)**** |
| Year of birth 2002 or later (ref. 1999 or earlier) | **1.922 (1.448-2.55)***** |
| **INCOME CATEGORY** |  |
| Percentile 71-90 (ref. 1-70) | 1.105 (0.806-1.515) |
| Percentile 91-100 (ref. 1-70) | 1.303 (0.962-1.765) |
| **ASSETS CATEGORY** |  |
| Percentile 41-80 (ref. 1-40) | **1.485 (1.118-1.972)**** |
| Percentile 81-100 (ref. 1-40) | **1.621 (1.208-2.175)**** |
| **NR. OF PARENTS ON WELFARE** |  |
| 1 or 2 parents (ref. 0) | 1.689 (0.889-3.207) |
| **NR. OF PARENTS WITH ANOTHER SOCIAL SERVICES INCOME** |  |
| 1 or 2 parents (ref. 0) | **0.667 (0.467-0.953)*** |
| **NR. OF PARENTS WITH BIG-REGISTRATION** |  |
| 1 or 2 parents (ref. 0) | 1.002 (0.764-1.314) |
| **NR. OF PARENTS WORKING IN PSVA EDUCATION** |  |
| 1 or 2 parents (ref. 0) | **1.433 (1.072-1.915)*** |
| **MIGRATION BACKGROUND** |  |
| TMSDI (ref. no migration background) | **0.572 (0.384-0.852)**** |
| Other (ref. no migration background) | 1.124 (0.854-1.479) |
| **URBANITY DEGREE** |  |
| Average (ref. (very) strongly urban) | 0.836 (0.658-1.062) |
| Weakly urban/not urban (ref. (very) strongly urban) | 0.919 (0.747-1.131) |

*p<.05; **p<0.01; ***p<0.001

Ref. = reference category; PSVA = Primary, Secondary or Vocational or Agrarian; TMSDI: Turkish, Moroccan, Surinamese, Dutch Caribbean, or Indonesian; Urbanity Degree of postal code of residential address in 2015. Results in **bold** are statistically significant (p<.05).
