# Supplementary material for: Inequality of opportunity in selection procedures limits diversity in higher education: An intersectional study of Dutch selective higher education programs
Source: PLoS One. 2023 Oct 13;18(10):e0292805. doi: 10.1371/journal.pone.0292805 (PMC10575509; doi:10.1371/journal.pone.0292805)
Supplement: S1 File — (DOCX) [file pone.0292805.s005.docx]

**S5: Intersectional acceptance rates based on background characteristics**

In this Supplemental Information, we show the acceptance rates of different groups of applicants, based on the variables that were significantly associated with receiving an offer of admission in the corresponding multivariable logistic regression models (see Supplemental Information 4).

Within individual clusters, groups are divided based on these characteristics until a division is no longer allowed due to at least one category having less than 10 persons. Statistics Netherlands regulations do not allow publication of frequencies <10.

Since a number of clusters had a large number of significant variables in their multivariable models, their figures had to be split up into multiple figures due to limited space on the page. In figures where this is the case, we have added an a, b, or c in the description of the figure. Example: SH1_2019a is an extension of the figure for SH1_2019.

# SH1_2019: From average to migration background to sex

Intersectional acceptance rates in the cluster of Allied Medical Care; Nursing; and Midwifery in 2019

## SH1_2019a: No migration background and male

Intersectional acceptance rates in the cluster of Allied Medical Care; Nursing; and Midwifery in 2019

Note: numbers do not always add up due to missing data

## SH1_2019b: No migration background and female

Intersectional acceptance rates in the cluster of Allied Medical Care; Nursing; and Midwifery in 2019

Note: numbers do not always add up due to missing data

## SH1_2019c: Other migration background and female

Intersectional acceptance rates in the cluster of Allied Medical Care; Nursing; and Midwifery in 2019

Note: numbers do not always add up due to missing data

# SH1_2020: From average to migration background to sex

Intersectional acceptance rates in the cluster of Allied Medical Care; Nursing; and Midwifery in 2020

## SH1_2020a: No migration background and female

Intersectional acceptance rates in the cluster of Allied Medical Care; Nursing; and Midwifery in 2020

Note: numbers do not always add up due to missing data

# SH2_2019: From average to migration background

Intersectional acceptance rates in the cluster of Dental Hygiene; Denturism; and Optometry in 2019

## SH2_2019a: No migration background

Intersectional acceptance rates in the cluster of Dental Hygiene; Denturism; and Optometry in 2019

Note: numbers do not always add up due to missing data

## SH2_2019b: Other migration background

Intersectional acceptance rates in the cluster of Dental Hygiene; Denturism; and Optometry in 2019

Note: numbers do not always add up due to missing data

SH2_2020
Intersectional acceptance rates in the cluster of Dental Hygiene; Denturism; and Optometry in 2020

Note: numbers do not always add up due to missing data

SH3_2019
Intersectional acceptance rates in the cluster of Biology and Medical Laboratory Research; Forensic Science; and Medical Imaging and Radiation Therapy in 2019

Note: numbers do not always add up due to missing data

# SH3_2020

Intersectional acceptance rates in the cluster of Biology and Medical Laboratory Research; Forensic Science; and Medical Imaging and Radiation Therapy in 2020

Note: numbers do not always add up due to missing data

# SH4_2019

Intersectional acceptance rates in the cluster of Physiotherapy; Psychomotoric Therapy/Psychomotricity; and Sport Studies in 2019

Note: numbers do not always add up due to missing data

# SH4_2020

Intersectional acceptance rates in the cluster of Physiotherapy; Psychomotoric Therapy/Psychomotricity; and Sport Studies in 2020

Note: numbers do not always add up due to missing data

# SH5_2019

Intersectional acceptance rates in the cluster of Creative Media and Game Technologies; Fashion & Textile Technologies; Industrial Design Engineering; and Art and Economics in 2019

Note: numbers do not always add up due to missing data

# SH5_2020: From average to migration background

Intersectional acceptance rates in the cluster of Creative Media and Game Technologies; Fashion & Textile Technologies; Industrial Design Engineering; and Art and Economics in 2020

Note: numbers do not always add up due to missing data

## SH5_2020a: No migration background

Intersectional acceptance rates in the cluster of Creative Media and Game Technologies; Fashion & Textile Technologies; Industrial Design Engineering; and Art and Economics in 2020

Note: numbers do not always add up due to missing data

## SH5_2020b: TMSDI migration background

Intersectional acceptance rates in the cluster of Creative Media and Game Technologies; Fashion & Textile Technologies; Industrial Design Engineering; and Art and Economics in 2020

Note: numbers do not always add up due to missing data

## SH5_2020c: Other migration background

Intersectional acceptance rates in the cluster of Creative Media and Game Technologies; Fashion & Textile Technologies; Industrial Design Engineering; and Art and Economics in 2020

Note: numbers do not always add up due to missing data

# SH6_2019

Intersectional acceptance rates in the cluster of Applied Psychology; Applied Biology; and Skin Therapy in 2019

Note: numbers do not always add up due to missing data

# SH6_2020

Intersectional acceptance rates in the cluster of Applied Psychology; Applied Biology; and Skin Therapy in 2020

Note: numbers do not always add up due to missing data

# SU1_2019

Intersectional acceptance rates in the cluster of Medicine in 2019

Note: numbers do not always add up due to missing data

# SU1_2020

Intersectional acceptance rates in the cluster of Medicine in 2020

Note: numbers do not always add up due to missing data

# SU2_2019

Intersectional acceptance rates in the cluster of Dentistry; and Pharmacy in 2019

Note: numbers do not always add up due to missing data

# SU2_2020

Intersectional acceptance rates in the cluster of Dentistry; and Pharmacy in 2020

Note: numbers do not always add up due to missing data

# SU3_2019

Intersectional acceptance rates in the cluster of Psychobiology; and Psychology in 2019

Note: numbers do not always add up due to missing data. In this cluster, there was a large amount of missing data on parental income category of applicants with an ‘Other migration background’.

# SU3_2020

Intersectional acceptance rates in the cluster of Psychobiology; and Psychology in 2020

Note: numbers do not always add up due to missing data

SU4_2019: From average to migration background

Intersectional acceptance rates in the cluster of Biomedical Sciences; Biomedical Engineering; and Clinical Technology in 2019

Note: numbers do not always add up due to missing data

## SU4_2019a: No migration background

Intersectional acceptance rates in the cluster of Biomedical Sciences; Biomedical Engineering; and Clinical Technology in 2019

Note: numbers do not always add up due to missing data

## SU4_2019b: TMSDI migration background

Intersectional acceptance rates in the cluster of Biomedical Sciences; Biomedical Engineering; and Clinical Technology in 2019

Note: numbers do not always add up due to missing data

## SU4_2019c: Other migration background

Intersectional acceptance rates in the cluster of Biomedical Sciences; Biomedical Engineering; and Clinical Technology in 2019

Note: numbers do not always add up due to missing data

SU4_2020

Intersectional acceptance rates in the cluster of Biomedical Sciences; Biomedical Engineering; and Clinical Technology in 2020

Note: numbers do not always add up due to missing data

# SU5_2019: From average to sex

Intersectional acceptance rates in the cluster of Biology; Biotechnology; Nutrition and Health; Veterinary Medicine; and Nanobiology in 2019

Note: numbers do not always add up due to missing data

## SU5_2019a: Male

Intersectional acceptance rates in the cluster of Biology; Biotechnology; Nutrition and Health; Veterinary Medicine; and Nanobiology in 2019

Note: numbers do not always add up due to missing data

## SU5_2019b: Female

Intersectional acceptance rates in the cluster of Biology; Biotechnology; Nutrition and Health; Veterinary Medicine; and Nanobiology in 2019

Note: numbers do not always add up due to missing data

# SU5_2020

Intersectional acceptance rates in the cluster of Biology; Biotechnology; Nutrition and Health; Veterinary Medicine; and Nanobiology in 2020

Note: numbers do not always add up due to missing data

# SU6_2019

Intersectional acceptance rates in the cluster of Artificial Intelligence; and Industrial Design in 2019

Note: numbers do not always add up due to missing data

# SU6_2020: no significant variables in the model

# SU7_2019

Intersectional acceptance rates in the cluster of Architecture, Urbanism & Building Sciences; Mechanical Engineering; Aerospace Engineering; Computer Science & Engineering; and Global Sustainability Science in 2019

Note: numbers do not always add up due to missing data

# SU7_2020

Intersectional acceptance rates in the cluster of Architecture, Urbanism & Building Sciences; Mechanical Engineering; Aerospace Engineering; Computer Science & Engineering; and Global Sustainability Science in 2020

Note: numbers do not always add up due to missing data. In this cluster, there was a large amount of missing data on parental assets percentile.

# SU8_2019

Intersectional acceptance rates in the cluster of Business Administration; International Business; International Business Administration; Tax Law; and Industrial Engineering & Management Science in 2019

Note: numbers do not always add up due to missing data. In this cluster, there was a large amount of missing data on urbanity degree of applicants with an ‘Other migration background’.

# SU8_2020

Intersectional acceptance rates in the cluster of Business Administration; International Business; International Business Administration; Tax Law; and Industrial Engineering & Management Science in 2020

Note: numbers do not always add up due to missing data. In this cluster, there was a large amount of missing data on urbanity degree of applicants with an ‘Other migration background’.

# SU9_2019

Intersectional acceptance rates in the cluster of International Relations and International Organization; Political Science; and Criminology in 2019

Note: numbers do not always add up due to missing data

# SU9_2020: From average to migration background to sex

Intersectional acceptance rates in the cluster of International Relations and International Organization; Political Science; and Criminology in 2019

Note: numbers do not always add up due to missing data

**As migration background and sex were the variables that showed the largest differences in odds of admission, we have also included figures that show the intersectional acceptance rates in the total dataset (2019 and 2020 combined), and separately for both years.**

# Total dataset in 2019 & 2020: From average to migration background to sex

Intersectional acceptance rates in the total dataset in 2019 and 2020

# Total dataset in 2019: From average to migration background to sex

Intersectional acceptance rates in the total dataset in 2019

# Total dataset in 2020: From average to migration background to sex

Intersectional acceptance rates in the total dataset in 2020
